# Eye Tracking of Occluded Self-Moved Targets: Role of Haptic Feedback and Hand-Target Dynamics

**DOI:** 10.1523/ENEURO.0101-17.2017

**Published:** 2017-07-03

**Authors:** Frederic Danion, James Mathew, J. Randall Flanagan

**Affiliations:** 1 Aix Marseille Univ, CNRS, Institut de Neurosciences de la Timone, Marseille, France; 2 Department of Psychology and Centre for Neurosciences Studies, Queen’s University, Kingston, ON, Canada

**Keywords:** Smooth pursuit, prediction, eye-hand coordination, internal models, hand-target mappings, target occlusion, haptic feedback

## Abstract

Previous studies on smooth pursuit eye movements have shown that humans can continue to track the position of their hand, or a target controlled by the hand, after it is occluded, thereby demonstrating that arm motor commands contribute to the prediction of target motion driving pursuit eye movements. Here, we investigated this predictive mechanism by manipulating both the complexity of the hand-target mapping and the provision of haptic feedback. Two hand-target mappings were used, either a rigid (simple) one in which hand and target motion matched perfectly or a nonrigid (complex) one in which the target behaved as a mass attached to the hand by means of a spring. Target animation was obtained by asking participants to oscillate a lightweight robotic device that provided (or not) haptic feedback consistent with the target dynamics. Results showed that as long as 7 s after target occlusion, smooth pursuit continued to be the main contributor to total eye displacement (∼60%). However, the accuracy of eye-tracking varied substantially across experimental conditions. In general, eye-tracking was less accurate under the nonrigid mapping, as reflected by higher positional and velocity errors. Interestingly, haptic feedback helped to reduce the detrimental effects of target occlusion when participants used the nonrigid mapping, but not when they used the rigid one. Overall, we conclude that the ability to maintain smooth pursuit in the absence of visual information can extend to complex hand-target mappings, but the provision of haptic feedback is critical for the maintenance of accurate eye-tracking performance.

## Significance Statement

The ability to predict visual consequences arising from our actions is central in daily activities. Here, we tested this ability by means of a task that required participants to track with the eyes a target that was occluded and whose motion was driven by the hand using simple or complex hand-target mappings both with and without haptic feedback. Our results showed that, despite a general drop in performance after target occlusion, smooth pursuit activity persisted under all conditions. Although haptic feedback was not critical under the simple mapping, it clearly improved performance under the complex one. We conclude that haptic feedback is critical to supplement vision when predicting the behavior of objects with complex dynamics.

## Introduction

The ability to anticipate sensory consequences resulting from self-initiated movement is central for current theories of motor control (Wolpert and Flanagan, 2001; Shadmehr et al., 2010; Wolpert et al., 2011). This ability can be demonstrated in various motor tasks ranging from object manipulation to eye–hand coordination. For instance, it is well established that smooth pursuit eye movements are substantially improved when the viewed target is moved by the subject’s hand in comparison to when it is moved by an external agent. This improvement is characterized by a higher gain in smooth pursuit (Mather and Lackner, 1975; Gauthier et al., 1988; Vercher et al., 1995), fewer saccades (Steinbach and Held, 1968; Steinbach, 1969; Angel and Garland, 1972; Mather and Lackner, 1975), and a shorter temporal lag between target and eye position (Steinbach and Held, 1968; Gauthier and Hofferer, 1976; Domann et al., 1989; Vercher et al., 1996). To account for these observations, it is proposed that the oculomotor system has access to an estimate of the current hand position through the combination of sensory feedback, arm efferent copy, and knowledge of hand-target dynamics (Scarchilli et al., 1999; Ariff et al., 2002). Overall, it is postulated that eye tracking profits from the ability to both predict future states of the limb (Ariff et al., 2002) and learn the mapping between hand actions and their visual consequences (Sailer et al., 2005).

The advantage of self-generated versus externally generated target motion in pursuit eye tracking is also seen when vision of the moving target is occluded. A large number of studies have examined eye movement behavior when vision of an externally driven moving target is transiently occluded (Bennett and Barnes, 2003, 2006; Madelain and Krauzlis, 2003; Orban de Xivry et al., 2008). A typical observation is that ∼200 ms after target blanking, performance in eye-tracking starts to deteriorate as indicated by a drop in smooth pursuit velocity and an increase in the contribution of catch-up saccades. Although only a few studies have investigated eye tracking when a self-moved target is temporarily masked, they indicate improved performance. In a seminal study, Gauthier and Hofferer (1976) explored the ability of participants to track a visual target that was moved by oscillating either the finger or the elbow. At some point the target was masked, and participants were asked to keep oscillating their limb while tracking the target as if it was still visible. Their results showed that participants were able to maintain smooth pursuit (albeit with a lower gain) over several successive cycles of movement lasting several seconds, which contrasts with the rapid decay of smooth pursuit when using an externally moved target. More recently, Berryhill et al. (2006), who investigated the ability to track a self-moved pendulum in the dark, also showed that participants could maintain smooth pursuit for several seconds (albeit with a low gain). Overall, the benefit of self-moving a target on smooth pursuit performance extends to situations in which the target is occluded.

The goal of the current study was to further investigate the ability of humans to track an invisible self-moved target, focusing on two key issues. First, we asked whether this ability extends to situations in which participants use a more complex mapping between hand and target motion. Second, we asked how this ability depends on receiving haptic feedback about the interaction between hand and target motion. To date, the contribution of haptic feedback to eye tracking has been (indirectly) investigated with deafferented patients under a simple hand-target mapping (Vercher et al., 1996), but not under a complex mapping and not when the target is occluded. To achieve these goals, we designed an experiment in which participants were asked to track a target on a screen whose animation was obtained by oscillating horizontally a grasped object attached to a lightweight robotic device. Two visual hand-target mappings were used, either a rigid one in which hand and target motion matched perfectly (simple dynamics) or a nonrigid one in which the target behaved as a mass attached to the hand by means of a spring (complex dynamics). Using the robotic device, haptic feedback congruent with the target dynamics could be provided or removed. Although previous studies have shown that people can learn to control nonrigid objects both when appropriate haptic feedback is provided (Dingwell et al., 2002, 2004; Mehta and Schaal, 2002; Nagengast et al., 2009) and when it is not (Mehta and Schaal, 2002; Mah and Mussa-Ivaldi, 2003), performance is typically improved by haptic feedback (Danion et al., 2012; Farshchiansadegh et al., 2016). Based on these results, we predicted that whereas eye tracking performance after target occlusion would be reduced when moving the nonrigid target in comparison to the rigid target, this deficit would be limited when haptic feedback was provided.

## Method

### Participants

Fourteen self-proclaimed right-handed participants (age, 22.2 ± 1.8 years; nine female) participated in this study. None of the participants had neurologic or visual disorders. They were naive as to the experimental conditions and hypotheses and had no previous experience of ocular motor testing. All participants gave written informed consent before the study. Each participant received $10 for participation. The Queens University ethics committee approved the experimental paradigm (2014-12-3-04), which complied with the Declaration of Helsinki.

### Apparatus

**Figure 1.**
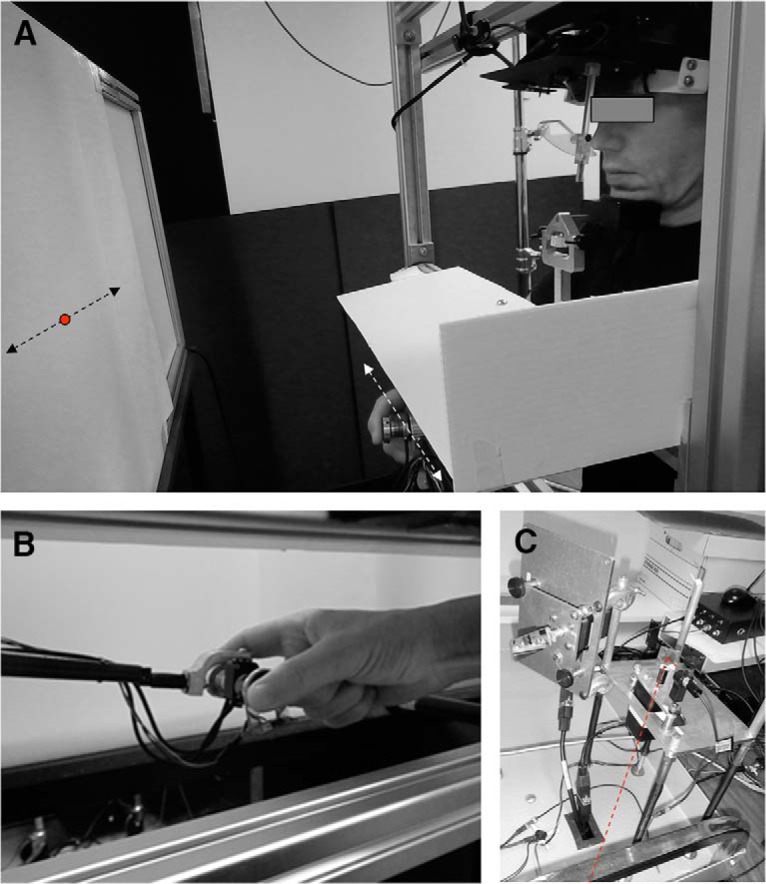
Photographs of the experimental setup. ***A***, Overview of the experimental setup. Red dot indicates target position when the laser is on. ***B***, The grasped object and the lightweight robotic device. ***C***, The laser and optical scanner. See text for more details.

The experimental setup is illustrated in Figure 1. Participants were comfortably seated facing a screen positioned in a frontal plane 57 cm away from the participant’s eye (Fig. 1*A*). Thus, at the center of the screen a target displacement of 1 cm corresponded to 1° in terms of visual angle. To minimize measurement errors, participants’ head movements were restrained by a chin rest and a padded forehead rest so that the eyes in primary position were directed toward the center of the screen. A bib was positioned under the participants’ chin to block vision of their hands. Participants controlled the position of a target on the screen by moving a grasped object attached to a lightweight robotic arm (Phantom Haptic Interface 3.0L, SensAble Technologies) in a frontal plane (Fig. 1*B*). When the target was at the center of the screen, the elbow and shoulder were comfortably positioned so that both hand and target lay in the participant’s midsagittal plane (Fig. 1*A*). Hand movements were recorded at a sampling rate of 1000 Hz with a resolution of 0.1 mm.

The target (filled red circle 0.5° in diameter) was projected on the screen using a laser beam (39080; Edmund Optics) moved by an optical scanner (MG350DT; General Scanning) servo-controlled by a PC (Fig. 1*C*). The delay in the servo-command was <1 ms. The optical scanner motion was restricted to one dimension so that the target moved only along the horizontal axis. An infrared video-based eye tracker (RK-726PCI pupil/corneal tracking system; ISCAN) was used to record the position of gaze of the left eye in the work plane at 400 samples/s. Before the experiment, we calibrated the output from the eye tracker by recording the raw eye positions as participants fixated on a grid composed of 25 known locations. The mean values during fixation intervals at each location were then used for converting offline raw eye tracker values to horizontal and vertical eye position in degrees of visual angle.

Two types of hand-target visual mapping were used. When participants had to move the rigid target, its motion was an exact replicate of the actual hand trajectory in the frontal plane: if the hand moved by 1 cm to the left, the target also moved by 1 cm to the left on the screen. When haptic feedback was implemented for the rigid target (Rigid-Hapt), interaction forces provided by the robotic device simulated the physical behavior of a 1-kg point mass. In the no haptic version of the rigid target (Rigid-NoHapt), the motors of the robotic device were simply turned off. When subjects had to move the nonrigid target (Spring), the visual target was simulated as a mass-spring object with the following properties: mass, 1 kg; stiffness, 40 N/m; damping, 1.66 N/m/s; resting length, 0 m. These values are about one-third of values used in previous studies investigating the manipulation of nonrigid objects (Dingwell et al., 2002, 2004; Nagengast et al., 2009; Danion et al., 2012; Landelle et al., 2016). The rational for decreasing object inertia was to prevent possible fatigue effects while keeping a 1-Hz resonance frequency as in other studies; the resonance frequency (*F*) of a mass-spring system depends on its mass (*m*) and its stiffness (*k*) such that

$$F=(\frac{1}{2\pi })*\sqrt{\left(\frac{k}{m}\right)}.$$

Depending on the experimental conditions, haptic feedback of the nonrigid target could be implemented in three different ways. First, haptic feedback could be provided such that it was congruent with the visual dynamics of the object (Spring-Hapt), meaning that the same parameters were used to simulate physical and visual behavior. Second, haptic feedback could be removed in the sense that motors of the robotic device were turned off (Spring-NoHapt). Third, haptic feedback could be incongruent with the visual dynamics of the object (Spring-DissHapt). In this case, we introduced a dissociation between the visual and haptic stiffness of the mass-spring (visual stiffness, 48 N/m; haptic stiffness, 32 N/m) while keeping mass and damping similar to previous values. This dissociation led to distinct resonance frequencies for the visual and haptic dynamics (1.25 vs. 0.8 Hz).

### Procedure

In all trials, participants were instructed to track as accurately as possible the target moving on the screen. The target motion was always driven by the subject’s hand. However, depending on the experimental condition, the visual mapping between hand movement and target motion could be either rigid or elastic, with either no haptic feedback, congruent haptic feedback, or incongruent haptic feedback. Overall, the following five experimental conditions were tested: Rigid-Hapt, Rigid-NoHapt, Spring-Hapt, Spring-NoHapt, and Spring-DissHapt. Each subject completed first a familiarization session in which they practiced the task with visual feedback followed by an experimental session in which, in each trial, visual feedback was initially present and then removed.

During the familiarization session, subjects were asked to perform random oscillatory movements to move the target (for a similar procedure, see Steinbach and Held, 1968; Angel and Garland, 1972). The underlying motivation was to favor the acquisition of knowledge about hand-target dynamics. Subjects were encouraged to use the whole extent of the screen (±20°) while making sure that the target did not fall outside the screen boundaries. To facilitate the production of random movements, a template was given before the training session. During demonstration trials, subjects did not move their hands and simply had to observe the replay of a trial performed by one of the experimenters under the same mapping condition. When subjects subsequently moved the target, we ensured that absolute target speed was comparable across conditions by encouraging subjects to maintain an average absolute target velocity close to 30°/s: this was possible by computing online mean absolute target velocity while the experimenters provided verbal feedback to the subject when necessary. This procedure ensured an overall mean target velocity of 30.2°/s with minimal changes across subjects (SD = 0.23°/s), experimental conditions (SD = 0.52°/s), and trials (SD = 0.80°/s). Each subject completed one block of 20 trials in each of the five experimental conditions. Each trial was 16 s long. Subjects were not explicitly informed about the nature of the mapping between their hand movement and the target motion before completing these experimental conditions. The order of blocks was randomized across subjects. Overall, a total of 100 trials (5 × 20 trials) were collected in this familiarization session.

Trials in the continuation session were similar to those in the familiarization session except that subjects were asked to produce target motion that was sinusoidal and the target was blanked after 7 s. Sinusoidal target motion was encouraged to simplify data analysis and allow comparison with previous work (Gauthier and Hofferer, 1976). A template with a target moving sinusoidally (period = 1000 ms; peak-to-peak amplitude = 15°; resulting in 30°/s mean absolute target velocity) was given before the continuation session. Subjects were encouraged to reproduce this pattern in the subsequent trials (effective mean period = 1.067 ms; effective mean amplitude = 17.3°). Each trial was 16 s long. Approximately 7 s after trial initiation, the target was removed from the screen (i.e., blanked) until the end of the trial. During the blanking period, subjects were instructed to keep oscillating the target while tracking it with their gaze as if it was still displayed on the screen. Each subject completed a block of three trials in each experimental condition. The order of the blocks was randomized across subjects. A total of 15 trials (5 × 3 trials) was collected during this experimental session. The overall duration of the familiarization with the experimental session averaged 60 min. Participants could request additional breaks at any time, but most of them only took the break offered between blocks.

### Data analysis

Because the stimuli were moving exclusively along the horizontal meridian, we focused our analyses on the horizontal component of eye movements. We performed a sequence of analysis to separate periods of smooth pursuit, saccades, and blinks from the raw eye position signals. The identification of the blinks was performed by visual inspection. This procedure led to the removal of ∼1% of eye recordings. Eye position time series were then low-pass filtered with a Butterworth (fourth order) using a cutoff frequency of 25 Hz. The resultant eye position signals were differentiated to obtain the velocity traces. The eye velocity signals were low-pass filtered with a cutoff frequency of 25 Hz to remove the noise from the numerical differentiation. The resultant eye velocity signals were then differentiated to provide the accelerations traces that we also low-pass filtered at 25 Hz to remove the noise. A dedicated Matlab script was run to identify saccades. This identification was based on the acceleration and deceleration peaks of the eye (>1500°/s^2^). Further visual inspection allowed to identify smaller saccades (<1°) that could not be identified automatically by our program. Based on these computations, periods of pursuit and of saccades were extracted.

During the experimental (continuation) trials, we distinguished regular pursuit from the periods of target occlusion (after ∼7 s). The first part and last part of each trial were analyzed separately. Each continuation trial was segmented into 1-s bins. The bin segmentation was set with respect to the initiation of target blanking. The first bin that followed the target blanking was named +1, and the bin just preceding the target blanking was named –1. We used the same policy to name the surrounding bins. A total of seven bins preceding the target blanking, and eight bins following target blanking could be reliably extracted from each trial. For each of these 1-s bins, we computed the same dependent variables. To assess baseline performance (i.e., in the presence of visual feedback), dependent variables were averaged across the five bins preceding target blanking (–5, –4, –3, –2, –1; bins –6 and –7 were discarded because stable performance was not reached yet). To assess performance during continuation (i.e., when target was invisible), dependent variables were averaged across the last five bins (4, 5, 6, 7, and 8).

To assess the participants’ ability to predict the dynamics of the target, we extracted the following dependent variables. First, we computed the mean absolute position error (PE) by averaging the absolute difference in position between the target and the eye over the whole trial, including periods of both saccades and smooth pursuit (note that excluding saccades from PE evaluation did not change our findings). Second, we computed the mean absolute velocity error (VE), i.e., the average absolute difference between the eye and target velocity. Note that although PE was evaluated over the whole trial (including periods of both saccades and smooth pursuit), VE was computed only during smooth pursuit periods. Third, as a gross index of temporal coupling, we computed the coefficient of correlation between eye and target position. Fourth, to evaluate more closely the temporal relationship between eye and target movements, we computed the lag between the two using a cross-correlation technique based on the eye and target position signal (a positive lag indicating the eye is lagging behind the target).

Finally, to assess the relative contribution of saccades and smooth pursuit, we computed for each trial the total distance traveled by the eye with saccades (Orban de Xivry et al., 2006) and then expressed this as a percentage of the total distance traveled by the eye using both saccades and smooth pursuit. To better characterize smooth pursuit, we computed the smooth pursuit gain by averaging the ratio between instantaneous eye and target velocities during phases of smooth pursuit (to avoid numerical instabilities, only situations where absolute target velocity was >10°/s were considered).

### Statistical analysis

Two-way ANOVA was used to assess the effects of target mapping and haptic feedback, but data before and after target blanking were analyzed separately. To subsequently investigate the effect of dissociating visual and haptic feedback, the condition Spring-DissHapt was contrasted with Spring-NoHapt and Spring-Hapt by means of one-way ANOVA. To obtain a normal distribution, *z*-score transformation was used for coefficients of correlation. Newman–Keuls corrections were used for *post hoc t* tests to correct for multiple comparisons. A conventional 0.05 significance threshold was used for all analyses.

## Results

### Typical trials

**Figure 2.**
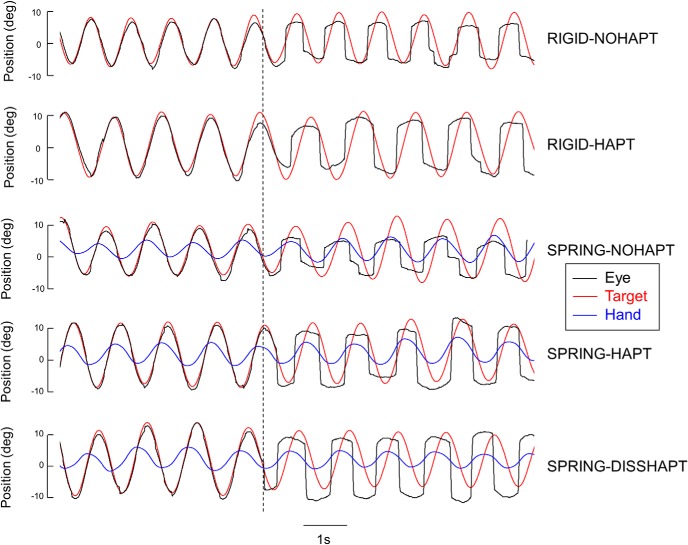
Typical trials performed by the same subject in each experimental condition. Vertical dotted line indicates the moment of target occlusion. Note the progressive drift in the temporal coupling between eye and target, and the larger contribution of saccades.

Figure 2 plots five typical trials performed by the same subject in each experimental condition. As can be seen, when the target was visible (first half of each trial), accurate smooth pursuit was observed in all five conditions. After target occlusion, although the rate and the amplitude of catch-up saccades increased, episodes of smooth pursuit were still observable (albeit with a lower gain). We also noticed a temporal drift between eye and target motion such that the eye started to lead the target, especially under the two conditions with the Spring mapping.

### Kinematics of target motion

Before target blanking, average group data indicated a mean period of target oscillation of 1065 ms, which was fairly close to the intended value (1000 ms). Blanking the target did not alter the mean period of oscillation (1061 ms). Concerning overall target movement amplitude, its mean value was 17.8° before target occlusion, which was slightly above the intended value (15°). After target blanking, this amplitude decreased by 10% under Rigid-NoHapt (*t*(26) = 2.11; *p* < 0.05), whereas it increased by 13% under Spring-NoHapt (*t*(26) = 3.37; *p* < 0.01).

### Eye motion: smooth pursuit versus saccades

**Figure 3.**
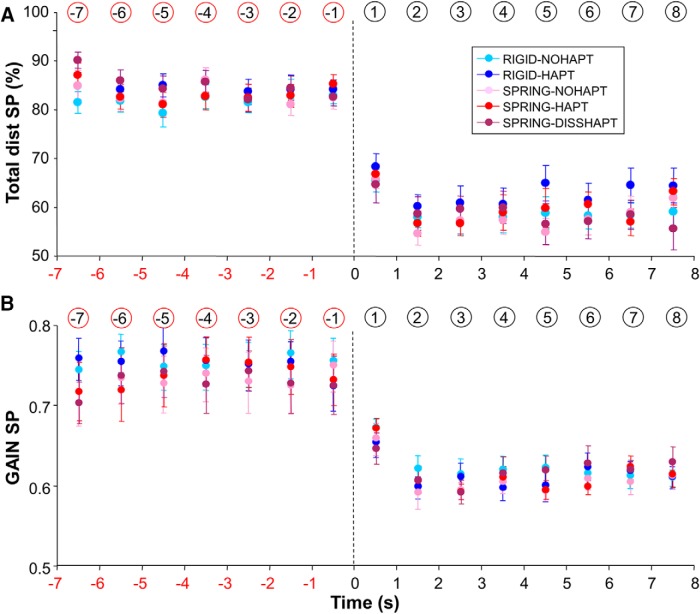
Contribution of saccades and smooth pursuit as a function of experimental condition and time in the vicinity of target occlusion. ***A***, Percentage of total distance covered by smooth pursuit. ***B***, Gain of smooth pursuit velocity. Error bars represent SE; vertical dotted lines denote the initiation of target occlusion; numbers circled (in red or black) denote the numbering of each bin (following the rationale described in Materials and Methods).

Figure 3*A* presents mean group data showing the relative contribution of smooth pursuit to eye tracking. This figure shows that before target occlusion, the percentage of total distance covered by the eye with smooth pursuit was high (∼83%), thereby confirming that the task was primarily performed using smooth pursuit eye movements. After target occlusion, this contribution decreased substantially (down to 60%; see also Fig. 2), but smooth pursuit remained the main contributor for eye movement. For both regular and blanked periods, ANOVAs showed no significant main effects of mapping and haptic feedback, as well as no interaction (*F*(1,13) < 3.75, *p* > 0.05). Further analyses showed that the increased contribution of saccades after target blanking was associated with a 30% increase in saccade rate and an approximate doubling of saccade amplitude (Fig. 2). Overall, despite an increase in the contribution of saccades, the key observation is that smooth pursuit activity persisted several seconds after target occlusion.

Regarding the gain of smooth pursuit, mean group data are presented in Figure 3*B*. As expected from previous studies (Gauthier and Hofferer, 1976; Berryhill et al., 2006), target occlusion was followed by a drop in smooth pursuit gain, which was observed in all conditions. Averaged across conditions, the gain decreased from 0.74 to 0.61, representing an 18% drop. Further analyses showed that, both before and after target occlusion, the gain was not significantly affected by mapping, haptic feedback, or the interaction between these factors (*F*(1,13) < 0.89, *p* > 0.05).

### Accuracy of eye tracking performance at the spatial level

**Figure 4.**
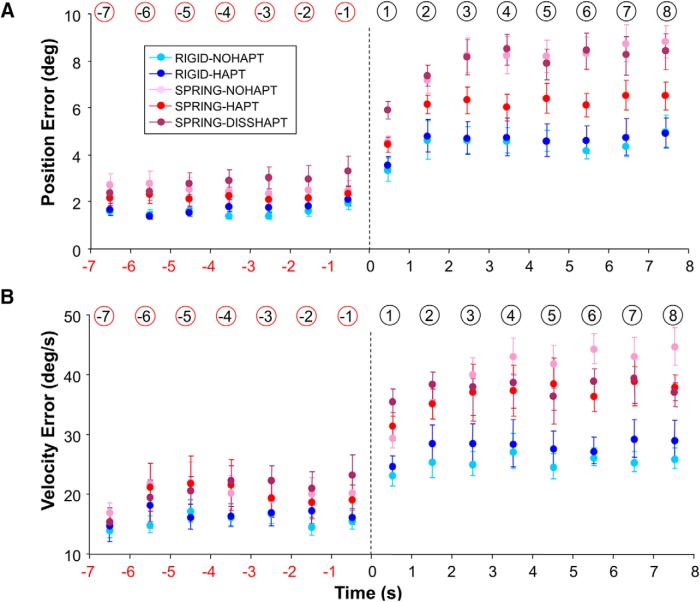
Spatial accuracy of eye tracking as a function of experimental condition and time in the vicinity of target occlusion. ***A***, Position error. ***B***, Velocity error between eye and target. Error bars represent SE; vertical dotted lines denote the initiation of target occlusion. Note how the detrimental effects of target blanking are reduced by the provision of haptic feedback under Spring. The numbers circled (in red or black) denote the numbering of each bin.

Having shown that smooth pursuit persists after target occlusion, we assessed its accuracy with respect to target motion. Figure 4 shows key parameters accounting for the spatial accuracy of eye tracking. Figure 4*A* presents the time course of PE across the five experimental conditions. Consider first the period before target occlusion. As expected, we found an effect of mapping (*F*(1,13) = 5.94; *p* = 0.03), consistent with the view that our task was more difficult under Spring than Rigid. On average, PE was 37% greater under Spring than Rigid (2.37 vs. 1.73°). However, we found no effect of haptic feedback (*F*(1,13) = 0.02; *p* = 0.88) and no interaction (*F*(1,13) = 2.56; *p* = 0.13). After target blanking, PE increased substantially in all experimental conditions. This time, the effects of haptic feedback (*F*(1,13) = 6.56, *p* = 0.02), mapping (*F*(1,13) = 86.02, *p* < 0.001), and their interaction (*F*(1,13) = 13.75; *p* = 0.003) were all significant. *Post hoc* analysis showed that whereas haptic feedback did not influence PE under Rigid, PE was 25% smaller under Spring-Hapt in comparison to Spring-NoHapt (6.36 vs. 8.49°; *p* < 0.001). Overall, this analysis of PE shows that, although haptic feedback had little influence on eye tracking accuracy as long as the target was visible, it became very important after target blanking under the Spring mapping.

Most of these observations were further supported by the analysis of the velocity error (VE; Fig. 4*B*). Indeed, when the target was visible, we also found an effect of mapping (*F*(1,13) = 7.15; *p* = 0.02) consistent with the view that tracking error was greater under Spring than Rigid (16.4 vs. 20.3°/s; 24%). Again we found no effect of haptic feedback (*F*(1,13) = 0.006; *p* = 0.94) as well as no interaction (*F*(1,13) = 0.16; *p* = 0.69). Furthermore, after target blanking, VE increased substantially in all conditions. However, once again this alteration was limited by the provision of haptic feedback under Spring but not under Rigid, as indicated by an interaction between haptic feedback and mapping (*F*(1,13) = 10.71, *p* = 0.006). *Post hoc* analysis confirmed that whereas VE values were similar in Rigid-Hapt and Rigid-NoHapt (28.4 vs. 25.9°/s; *p* = 0.17), VE was 13% smaller in Spring-Hapt compared with Spring-NoHapt (38.0 vs. 43.5°/s; *p* < 0.001).

### Accuracy of eye tracking performance at the temporal level

**Figure 5.**
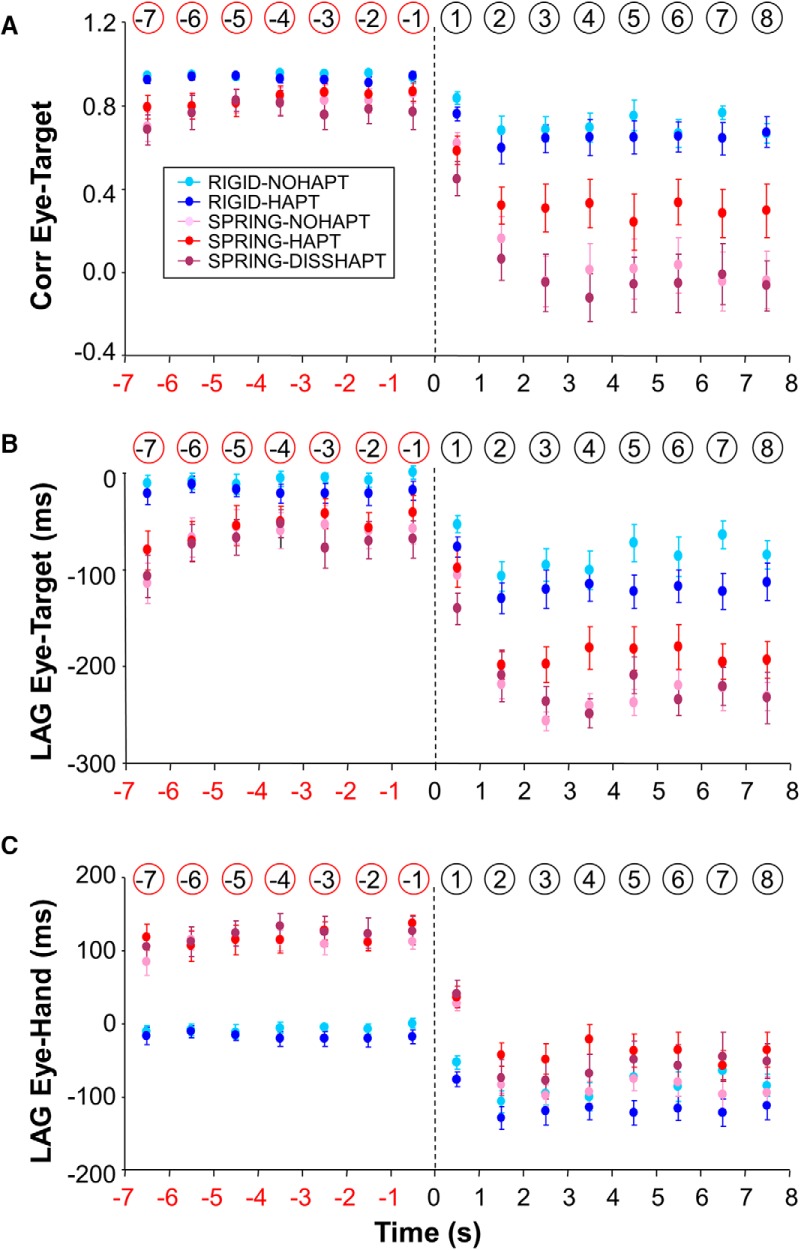
Temporal accuracy of eye tracking as a function of experimental condition and time in the vicinity of target occlusion. ***A***, Coefficient of correlation between eye and target. ***B***, Temporal lag between eye and target. A negative lag indicates that the eye precedes the target. ***C***, Temporal lag between eye and hand. A negative lag indicates that the eye precedes the hand. Error bars represent SE; vertical dotted lines denote the initiation of target occlusion; numbers circled (in red or black) denote the numbering of each bin. Note how the detrimental effects of target blanking are reduced by the provision of haptic feedback under Spring.

As for spatial accuracy, temporal accuracy of eye tracking decreased after target occlusion. To investigate the temporal coordination between eye and target, we first present the correlation coefficient between the two corresponding position signals (Fig. 5*A*). When the target was visible, we found no significant main effect of mapping and haptic feedback as well as no interaction (F(1,13)<1.39; *p* > 0.26). In contrast, when the target was blanked, we found a mapping × haptic feedback interaction (*F*(1,13) = 54.88; *p* < 0.001) such that *R* values were similar under the two Rigid conditions (NoHapt 0.71 vs. Hapt 0.65; *p* = 0.12), but were greater under Spring-Hapt compared with Spring-NoHapt (0.30 vs. –0.01; *p* < 0.001). Overall, this analysis adds further evidence that haptic feedback was helpful when the target was occluded, but only under the Spring mapping.

To further examine this alteration in temporal accuracy, Figure 5*B* and *C* present the temporal lag between eye and target and between eye and hand, respectively. Importantly, one should keep in mind that, in contrast to Rigid, where hand and target motion are inherently synchronized, there is a temporal delay between hand and target motion under Spring. Because of the mass-spring dynamics, the motion of the target lagged hand motion by ∼200 ms (bottom three rows in Fig. 2). Mean group lags—combining data from pre- and postocclusion—were 182, 171, and 245 ms, respectively, in Spring-NoHapt, Spring-Hapt, and Spring-DissHapt (in the latter condition, the delay was greater because of the lower stiffness of the visual spring).

Concerning the temporal relationship between eye and target (Fig. 5*B*), we found that when the target was visible, eye and target were rather well synchronized under Rigid (mean lag, –13 ms) whereas a clear eye lead was seen under Spring (mean lag, –53 ms). This difference in eye behavior was corroborated by a main effect of mapping (*F*(1,13) = 11.06; *p* = 0.005). Unexpectedly, when the target was blanked, the eye began to lead even more on the target, a phenomenon observed in all conditions albeit with different intensity. First, this effect was more pronounced under Spring than Rigid (–208 vs. –100 ms; *F*(1,13) = 73.94; *p* < 0.001). Second, we also found an interaction between mapping and haptic feedback (*F*(1,13) = 12.95; *p* = 0.003), such that a smaller phase lead was observed under Spring-Hapt than Spring-NoHapt (–186 vs. –230 ms; *p* < 0.05). In contrast, the phase lead was similar under both Rigid conditions (*p* = 0.14). Overall haptic feedback appeared helpful in limiting the temporal drift of the eye induced by the target blanking under Spring.

To provide a better understanding of eye–hand temporal coordination, we present the temporal lag between eye and hand (Fig. 5*C*). As expected when the target was visible, there was a clear effect of mapping (*F*(1,13) = 232.3; *p* < 0.001). Whereas under Spring the eye lagged behind the hand (mean lag, 117 ms), the eye and hand were synchronized under Rigid (mean lag, –12 ms). After target blanking, the timing between eye and hand changed substantially, with the eye leading the hand under all conditions. However, the magnitude of this eye lead depended on both mapping and haptic feedback, as revealed by an interaction between the two factors (*F*(1,13) = 50.06; *p* < 0.001). *Post hoc* analysis indicated that in the absence of haptic feedback, the lead of the eye became similar under Rigid-NoHapt and Spring-NoHapt (–82 and –88 ms, respectively; *p* = 0.46), despite substantial differences before target blanking. In contrast, when haptic feedback was provided, the differences in eye–hand lag persisted between the two mappings (Rigid-Hapt, –118 ms; Spring-Hapt, –38 ms; *p* < 0.001). Overall, this analysis reinforces the view that the provision of haptic feedback is important for eye–hand coordination after target masking.

### Dissociation between haptic and visual feedback

Many dependent variables (PE, VE, eye–target correlation, and eye–target lag) showed that the provision of haptic feedback was helpful in reducing the detrimental effects of target blanking under the Spring mapping. For each of these variables, we ran an additional ANOVA that compared the three Spring conditions, namely Spring-DissHapt, Spring-NoHapt, and Spring-Hapt. Except for VE, we found in all cases a significant difference across these three conditions (*F*(2,26) > 4.15; *p* < 0.05). *Post hoc* analyses consistently showed a lack of difference between Spring-DissHapt and Spring-NoHapt, while showing reliably a difference between Spring-DissHapt and Spring-Hapt (*p* < 0.05). Overall these additional analyses show that when haptic feedback was not congruent with visual dynamics of the target, the benefit provided by haptic feedback was lost.

### Comparison between a self-moved and an externally moved target

**Figure 6.**
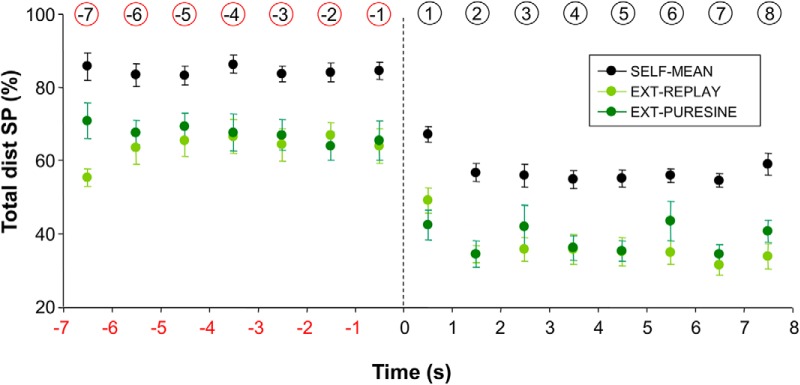
Percentage of total distance covered by smooth pursuit as a function of experimental condition and time in the vicinity of target occlusion. This figure compares the performance of two groups of participants that tracked either a self-moved target or an externally moved one. See text for more details. Error bars represent SE; vertical dotted line denotes the initiation of target occlusion; numbers circled (in red or black) denote the numbering of each bin.

One major conclusion drawn from this study is that, after target occlusion, smooth pursuit remains the major contributor of eye motion, with ∼60% of the total distance covered by the eye. An implicit assumption is that the contribution of smooth pursuit would have been smaller for an externally moved target. To test this assumption, we tested seven new participants using a similar paradigm, except this time the motion of the target was preprogrammed and the hand was immobile. After a familiarization session, each participant was tested successively with pure sinusoidal trajectories (frequency, 1 Hz; amplitude, 15°) and target trajectories taken from a randomly selected participant of the previous experiment (with a different previous participant matched to each new participant). As in the previous conditions, the target was blanked after 7 s, and each participant performed a block of three trials in each condition. Figure 6 shows the results of this control experiment separately for the pure sinusoidal trajectories (Ext-PureSine) and the playback trajectories (Ext-Replay). For comparison purposes, we also present the mean performance of the seven participants whose trajectories were used for playback (Self-Mean). Although there was no significant difference between Ext-PureSine and Ext-Replay (*F*(1,6) = 2.01; *p* = 0.21), the contribution of smooth pursuit was always smaller in Ext compared with Self (*F*(1,6) = 41.65; *p* < 0.001). This control experiment shows that for both visible and occluded targets, there is a greater contribution of smooth pursuit when tracking a self-moved target compared with an externally moved one. Those results extend earlier observations made in the context of nonoccluded targets (Steinbach and Held, 1968; Landelle et al., 2016) in the sense that they are also valid for occluded targets.

## Discussion

The goal of this study was to investigate the ability of humans to track with their eyes a self-moved target in the absence of visual feedback. Specifically, we asked whether haptic feedback could ameliorate the effects of removing visual feedback when the dynamics relating hand and target motion were either simple or complex. Our experiment resulted in four key findings. First, we found that participants were able to maintain smooth pursuit activity after target occlusion, even under a complex hand-target mapping. Second, although largely expected, target occlusion was detrimental for the accuracy of eye tracking under all conditions. Third, the detrimental effects of target occlusion on eye tracking depended to a large extent on both the target dynamics and the availability of haptic feedback. Specifically, although haptic feedback did not provide much benefit to eye tracking under the Rigid mapping, it limited the detrimental effects of target occlusion under the Spring mapping. Finally, when haptic and visual feedback were dissociated (i.e., incongruent), the benefit of haptic feedback (seen under Spring-Hapt) was no longer observed. We now discuss in more detail these findings and their implications.

### Maintenance of smooth pursuit after target occlusion

The current study showed that participants can maintain reliable smooth pursuit activity for several seconds after the masking of a self-moved target, which further reinforces the view that retinal slip is not the only input to the smooth pursuit control system (Gauthier and Hofferer, 1976; Vercher et al., 1996; Berryhill et al., 2006). However, the contribution of smooth pursuit to tracking was reduced when the target was occluded (switching from 83% to 60%) and the smooth pursuit gain decreased (from 0.74 to 0.61). These detrimental effects are consistent, at least qualitatively, with two earlier seminal studies. In the study of Jordan (1970) smooth pursuit contribution dropped from 98% to 38% when the target was occluded. In the study of Gauthier and Hofferer (1976), the gain of smooth pursuit dropped from ∼1 to 0.7, and their Fig. 4 also speaks for a decreased contribution of smooth pursuit after target occlusion. Because both studies only used conventional hand-target mappings (i.e., rigid), one novel contribution of our study is to extend the ability of participants to maintain smooth pursuit activity under more complex mappings. In a recent study by Landelle et al. (2016), target occlusions were also investigated under a Spring mapping, but their duration (400 ms) was less challenging than in the current experiment. Thanks to longer periods of target occlusion (7 s) in our study, we showed that at least 2 s were necessary to stabilize eye behavior (Figs. 3, 4, and 5).

### The role of hand-target mapping and haptic feedback

Smooth pursuit activity can be maintained consistently after target occlusion, but the accuracy of eye tracking was altered by this procedure. Although this was largely expected (Berryhill et al., 2006), this alteration depended both on the type of hand-target mapping and the availability of haptic feedback. More specifically, all our analyses of spatial and temporal accuracy (Figs. 4 and 5) indicated an interaction between mapping and haptic feedback. In all cases, haptic feedback was helpful to circumvent the detrimental effects of target occlusion under the Spring mapping but not under the Rigid one. For instance, when maneuvering the spring target, the provision of haptic feedback led to a reduction in PE and VE of 25% and 13%, respectively.

The temporal coordination between eye and target was also altered by the occlusion, but the largest changes were observed under the Spring mapping, with the eye starting to substantially lead the target (Fig. 5*B*) and even the hand, a phenomenon that was also observed under the Rigid mapping (Fig. 5*C*). In the absence of haptic feedback, this temporal drift was so large under Spring that ultimately the eye-hand lag became similar under Rigid, suggesting that participants failed to maintain a representation of the spring linking target and hand motion. In contrast, when haptic feedback was provided, the drift was smaller (–20%), allowing to maintain different eye–hand timing under Spring and Rigid. We conclude that the provision of haptic feedback under Spring was helpful in maintaining the initial coordination between eye, hand, and target.

More generally, the contribution of haptic feedback is very context specific. When manipulating the rigid target both with and without occlusion, haptic feedback did not influence eye–hand coordination. In that sense, our results are consistent with earlier observations made by Vercher et al. (1996), who reported that, under a simple (i.e., rigid) mapping, deafferented patients did not differ from control participants when tracking a nonoccluded target. Moreover, when manipulating the nonoccluded spring target, we did not find any obvious contribution of haptic feedback. However, as soon as the spring target was occluded, a contribution of haptic feedback emerged. Overall, the results suggest that when participants maneuver familiar objects, haptic feedback is unnecessary to drive their eye motion (visual information and hand efference copy being sufficient). In contrast, when conditions become more challenging, such as with an unfamiliar object (i.e., with complex dynamics) and in the absence of visual feedback, haptic feedback can provide a critical input for eye tracking.

### Dissociation between haptic and visual feedback

Results showed that when the haptic and visual dynamics were dissociated, the benefit of haptic feedback under target occlusion was lost; namely, eye tracking performance became as poor as with no haptic feedback. In principle, participants could have learned, before target occlusion, the rather arbitrary mapping between haptic and visual feedback and use dit to predict target motion after target occlusion. However, our results indicate that this did not occur. Alternatively, following target occlusion, participants could have interpreted that haptic feedback was congruent with visual target motion (e.g., as in the Spring-Hapt or Rigid-Hapt conditions) and used this mapping to predict target motion. However, this is unlikely, because eye tracking performance should have become worse than with no haptic feedback. Instead, it seems that participants simply ignored the incongruent haptic feedback, presumably after learning that it was not consistent with visual feedback during the preblanking phase. All in all, this suggests that participants can flexibly rely on haptic feedback when it is helpful for the task, but can also ignore it when it is potentially harmful for the task.

### Eye lead after target occlusion

Under all experimental conditions, eye motion was shifted forward in time after the target was occluded. Averaged across conditions, the mean lead of the eye over the hand was 76 ± 31 ms. Similarly, Gauthier and Hofferer (1976) reported that “the eye led the finger by an averaged 60-ms delay in all tests involving tracking of an imaginary target actively moved by the finger.” They suggested that this lead arises because hand and eye movements have different response times to motor commands, mainly because the eye has considerably lower inertia and is driven by relatively stronger muscles. It was proposed that in total darkness, since there is no more need to compensate for this asymmetry in motor systems, a phase lead of the eye would emerge. More recently, a rather similar phenomenon was observed when participants were asked to try to look at the perceived position of their hand during unseen reaching movements (Ariff et al., 2002). In that context, saccades provided an unbiased estimate of hand position at *t* + 200 ms. Namely, participants initiated saccades that landed close to where their hand would be 200 ms later. The authors interpreted that finding as evidence that the brain uses a forward model allowing the eye to estimate the future hand position in real time as movement unfolds (Scarchilli et al., 1999). Although attractive, this scheme does not account for the eye lead when participants are explicitly required to track their hand. A possible reason for this behavior may stem from natural eye–hand coordination during object manipulation. Indeed, when participants are asked to transport an object, their gaze is typically leading the hand (Johansson et al., 2001; Sailer et al., 2005). Specifically, participants make so-called proactive saccades, meaning that their gaze is directed toward the location where they plan to bring the object. We propose that when the target was occluded, although our participants were explicitly required to track the current position of the target, they might experience difficulties refraining from this proactive gaze behavior.

## Conclusions

Overall, we conclude that the ability to maintain smooth pursuit in the absence of visual information extends to complex hand-target mappings, but the provision of haptic feedback is critical for the maintenance of accurate eye-tracking performance. More generally, this study extends the view that haptic feedback is critical not only for manipulating nonrigid objects efficiently (Sternad et al., 2001; Huang et al., 2006; Danion et al., 2012), but also to coordinate proficiently eye and hand actions.

## Synthesis

Reviewing Editor: Tatiana Pasternak, University of Rochester Decisions are customarily a result of the Reviewing Editor and the peer reviewers coming together and discussing their recommendations until a consensus is reached. When revisions are invited, a fact-based synthesis statement explaining their decision and outlining what is needed to prepare a revision will be listed below. The following reviewer(s) agreed to reveal their identity: Jing Chen, Edward Freedman

SYNTHESIS

The work described in the manuscript examined the role of haptic feedback on the ability of subjects to track occluded visual targets. The paper reports that haptic feedback affects smooth pursuit eye movements but that this effect is context-specific. The paper provides new information about role of haptic feedback in smooth pursuit which is of great interest to the field. The reviewers commended the clarity of the paper and of the results. However, they raised a number of issues that must be addressed before the manuscript can be accepted for publication in eNeuro. These are summarized below.

1. Address the question raised by Reviewer 1 concerning the baseline control condition that will make it possible to evaluate the change in the contribution of smooth pursuit from 83% to 60%.

2. Address the possibility raised by Reviewer 1 that haptic feedback may have helped SPRNG mapping because the subjects were less familiar with that type of movement by checking for potential practice effects

3. Please cite and discuss the existing literature on the role of haptic feedback

4. Address the point made by Reviewer 2 about presenting discrete data as histograms rather than as a continuous line (see figs 3,4,5)

5. In response to reviewer 2, provide a clearer explanation of Fig 1. Is the target in Fig 1A actually visible?

6. As suggested by Reviewer 2, try to shorten the Discussion.

7. Please correct the wording and errors highlighted by both reviewers (at the end of their respective reviews).
